# Estimating drivers of cell state transitions using gene regulatory network models

**DOI:** 10.1186/s12918-017-0517-y

**Published:** 2017-12-13

**Authors:** Daniel Schlauch, Kimberly Glass, Craig P. Hersh, Edwin K. Silverman, John Quackenbush

**Affiliations:** 1 Department of Biostatistics and Computational Biology, Dana-Farber Cancer Institute and Department of Biostatistics, Harvard TH Chan School of Public Health, Boston, 02115 MA USA; 2 Channing Division of Network Medicine, Brigham and Women’s Hospital, Boston, 02115 MA USA; 3 Department of Medicine, Harvard Medical School, Boston, 02115 MA USA; 4 Pulmonary and Critical Care Division, Brigham and Women’s Hospital, Boston, 02115 MA USA

**Keywords:** Gene regulatory network inference, Chronic obstructive pulmonary disease, Genomics

## Abstract

**Background:**

Specific cellular states are often associated with distinct gene expression patterns. These states are plastic, changing during development, or in the transition from health to disease. One relatively simple extension of this concept is to recognize that we can classify different cell-types by their active gene regulatory networks and that, consequently, transitions between cellular states can be modeled by changes in these underlying regulatory networks.

**Results:**

Here we describe **MONSTER**, MOdeling Network State Transitions from Expression and Regulatory data, a regression-based method for inferring transcription factor drivers of cell state conditions at the gene regulatory network level. As a demonstration, we apply MONSTER to four different studies of chronic obstructive pulmonary disease to identify transcription factors that alter the network structure as the cell state progresses toward the disease-state.

**Conclusions:**

We demonstrate that MONSTER can find strong regulatory signals that persist across studies and tissues of the same disease and that are not detectable using conventional analysis methods based on differential expression. An R package implementing MONSTER is available at github.com/QuackenbushLab/MONSTER.

**Electronic supplementary material:**

The online version of this article (doi:10.1186/s12918-017-0517-y) contains supplementary material, which is available to authorized users.

## Author summary

Biological states are characterized by distinct patterns of gene expression that reflect each phenotype’s active cellular processes. Driving these phenotypes are gene regulatory networks in which transcriptions factors control when and to what degree individual genes are expressed. Phenotypic transitions, such as those that occur when disease arises from healthy tissue, are associated with changes in these networks. MONSTER is a new approach to understanding these transitions. MONSTER models phenotypic-specific regulatory networks and then estimates a “transition matrix” that converts one state to another. By examining the properties of the transition matrix, we can gain insight into regulatory changes associated with phenotypic state transition. We demonstrate the power of MONSTER by applying it to data from four independent studies of chronic obstructive pulmonary disease and find a robust set of transcription factors that help explain the development of the disease.

## Background

Cell state phenotypic transitions, such as those that occur during development, or as healthy tissue transforms into a disease phenotype, are fundamental processes that operate within biological systems. Understanding what drives these transitions, and modeling the processes, is one of the great open challenges in modern biology. One way to conceptualize the state transition problem is to imagine that each phenotype has its own characteristic gene regulatory network, and that there are a set of processes that are either activated or inactivated to transform the network in the initial state into one that characterizes the final state. Identifying those changes could, in principle, help us to understand not only the processes that drive the state change, but also how one might intervene to either promote or inhibit such a transition.

Each distinct cell state consists of a set of characteristic processes, some of which are shared across many cell-states (“housekeeping” functions) and others which are unique to that particular state. These processes are controlled by gene regulatory networks in which transcription factors (and other regulators) moderate the transcription of individual genes whose expression levels, in turn, characterize the state. One can represent these regulatory processes as a directed network graph, in which transcription factors and genes are nodes in the network, and edges represent the regulatory interactions between transcription factors and their target genes. A compact representation of such a network, with interactions between *m* transcription factors and *p* target genes, is as a binary *p*×*m* “adjacency matrix”. In this matrix, a value of 1 represents an active interaction between a transcription factor and a potential target, and 0 represents the lack of a regulatory interaction.

When considering networks, a cell state transition is one that transforms the initial state network to the final state network, adding and deleting edges as appropriate. Using the adjacency matrix formalism, one can think of this as a problem in linear algebra in which we attempt to find an *m*×*m* “transition matrix” **T**, subject to a set of constraints, that approximates the conversion of the initial network’s adjacency matrix **A** into the final network’s adjacency matrix **B**, or

1 $$ \mathbf{B=AT}  $$

In this model, we describe the differences between cell states with a lower dimensional transition matrix. This matrix allows for the estimation of a relatively smaller number of parameters which focus on larger systemic shifts in regulatory behavior by TFs. Intuitively, one might recognize that the true transition matrix between identical network states is the identity matrix because the diagonal elements of **T** map network edges to themselves. Deviations from this identity, specifically the observation of meaningful non-zero values off of the diagonal, provide evidence of changes in regulatory network configuration for TFs between states.

**Fig. 1:**
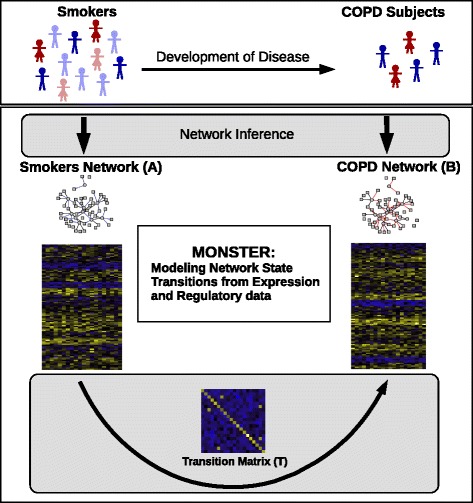
Overview of the MONSTER approach, as applied to the transition between smokers and those suffering from chronic obstructive pulmonary disease (COPD). MONSTER’s approach seeks to find the *TF*×*TF* transition matrix that best characterizes the state change in network structure between the initial and final biological conditions. Subjects are first divided into two groups based on whether they have COPD or are smokers that have not yet developed clinical COPD. Network inference is then performed separately on each group, yielding a bipartite adjacency matrix connecting transcription factors to genes. Finally, a transition matrix is computed which characterizes the conversion from the consensus Smokers Network to the COPD Network

While this framework, as depicted in Fig. 1, is intuitive, it is a bit simplistic in that we have cast the initial and final states as discrete. However, the model can be generalized by recognizing that any phenotype we analyze consists of a collection of individuals, all of whom have a slightly different manifestation of the state, and therefore a slightly different active gene regulatory network. Practically, what that means is that for each state, rather than having a network model with edges that are either “on” or “off,” a phenotype should be represented by a network in which each edge has a weight that represents an estimation of its presence across the population. In other words, the initial and final state adjacency matrices are not comprised of 1’s and 0’s, but of continuous variables that estimate population-level regulatory network edge-weights. Consequently, the problem of calculating the transition matrix is generalized to solving **B**=**A** **T**+**E**, where **E** is an *p*×*m* error matrix. In this expanded framework, modeling the cell state transition remains equivalent to estimating the appropriate transition matrix **T**, and then identifying state transition drivers based on features of that matrix.

## Methods

### MONSTER: MOdeling Network State Transitions from Expression and Regulatory data

The MONSTER algorithm models the regulatory transition between two cellular states in three steps: (1) Inferring state-specific gene regulatory networks, (2) modeling the state transition matrix, and (3) computing the transcription factor involvement.

**Inferring state-specific gene regulatory networks:** Before estimating the transition matrix, **T**, we must first estimate a gene regulatory starting point for each state. While there have been many methods developed to infer such networks [1–7], we have found the bipartite framework used in PANDA [8] to have features that are particularly amenable to interpretation in the context of state transitions. PANDA begins by using genome-wide transcription factor binding data to postulate a network “prior”, and then uses message-passing to integrate multiple data sources, including state-specific gene co-expression data.

Motivated by PANDA, we developed a highly computationally efficient, classification-based network inference method that uses common patterns between transcription factor targets and gene co-expression to estimate edges and to generate a bipartite gene regulatory network connecting transcription factors to their target genes.

This approach is based on the simple concept that genes affected by a common transcription factor are likely to exhibit correlated patterns of expression. To begin, we combine gene co-expression information with information about transcription factor targeting derived from sources such as ChIP-Seq or sets of known sequence binding motifs found in the vicinity of genes. The process of building prior networks to use as input to MONSTER may be complex, but our tool is agnostic to the source of this input. Users of MONSTER should use domain specific knowledge to generate an appropriate prior network.

We then calculate the direct evidence for a regulatory interaction between a transcription factor and gene, which we define as the squared partial correlation between a given transcription factor’s gene expression, *g*_*i*_, and the gene’s expression, *g*_*j*_, conditional on all other transcription factors’ gene expression:

$$\hat{d}_{i,j}=cor\left(g_{i},g_{j}|\left\{ g_{k}:k\ne i,k\in\mathbf{TF}_{\mathbf{j}}\right\} \right)^{2}, $$

where *g*_*i*_ is the gene which encodes the transcription factor *TF*_*i*_, *g*_*j*_ is any other gene in the genome, and **TF**_*j*_ is the set of gene indices corresponding to known transcription factors with binding site in the promoter region of *g*_*j*_. The correlation is conditioned on the expression of all other potential regulators of *g*_*j*_ based on the transcription factor motifs associated with *g*_*j*_. The direct evidence is motivated by the idea that changes in transcription factor expression may lead to similar changes in in target gene expression. The coexpression of co-targeted genes is long established in the literature [9, 10], and evidence also points to the coexpression of transcription factor genes with targets of that transcription factor [11, 12]. Moreover, studies across multiple tissues have shown widely varying expression of transcription factor genes, indicating that this expression can be used to predict their regulatory involvement [13]. Naturally, transcription factor behavior depends on many factors, including those that occur after translation. However, it makes intuitive sense that the mRNA abundance of a gene for a transcription factor should correlate with target genes to some degree.

Next, we fit a logistic regression model which estimates the probability of each gene, indexed *j*, being a motif target of a transcription factor, indexed *i*, based on the expression pattern across the *n* samples across *p* genes in each phenotypic class:

$$logit\left(P\left[\mathbf{M}_{i,j}=1\right]\right)=\beta_{0,i}+\beta_{1,i}g_{j}^{(1)}+\dots+\beta_{N,i}g_{j}^{(N)} $$

$$\hat{\theta}_{i,j}=\frac{e^{\hat{\beta}_{0,i}+\hat{\beta}_{1,i}g_{j}^{(1)}+\dots+\hat{\beta}_{N,i}g_{j}^{(N)}}}{1+e^{\hat{\beta}_{0,i}+\hat{\beta}_{1,i}g_{j}^{(1)}+\dots+\hat{\beta}_{N,i}g_{j}^{(N)}}} $$

where the response **M** is a binary *p*×*m* matrix indicating the presence of a sequence motif for the *i* ^*th*^ transcription factor in the vicinity of each of the *j* ^*th*^ gene. And where $g_{j}^{(k)}$ represents the gene expression measured for sample *k* at gene *j*. Thus, the fitted probability $\hat {\theta }_{i,j}$ represents our estimated indirect evidence. Combining the scores for the direct evidence, $\hat {d}_{i,j}$, and indirect evidence, $\hat {\theta }_{i,j}$, via weighted sum between each transcription factor-gene pair yields estimated edge-weights for the gene regulatory network. We score each gene according to the strength of indirect evidence for a regulatory response to each of the transcription factors and combine this with the direct evidence of regulation. Combining our measures of direct and indirect evidence presents some challenges. Though both are bounded by [0,1] their interpretations are quite different. The direct evidence can be considered in terms of its conditional gene expression *R* ^2^ between nodes, while the indirect evidence is interpreted as an estimated probability. Therefore, we use a non-parametric approach to combine evidence. Specifically, the targets of each transcription factor are ranked and combined as a weighted sum, $w_{i,j}=\left (1-\alpha \right)\left [rank\left (\hat {d}_{i,j}\right)\right ]+\alpha \left [rank\left (\hat {\theta }_{i,j}\right)\right ]$, where *α* is a constant bounded between [0,1]. Our choice of the weight is by default *α*=0.5, corresponding to an equal contribution of direct and indirect evidence. This parameter could be adjusted if the context of a study involved reason to prefer one source of evidence over the other (see Supporting Information).

Applying this approach to gene expression data from two distinct phenotypes results in two *p*×*m* gene regulatory adjacency matrices, one for each phenotype. These matrices represent estimates of the targeting patterns of the *m* transcription factors onto the *p* genes. This network inference algorithm finds validated regulatory interactions in *Escherichia coli* and Yeast (*Saccharomyces cerevisiae*) data sets (see Supporting Information).

**Modeling the state transition matrix:** Many methods have been developed for inferring gene regulatory networks, but more recent work has been proposed for estimating gene regulatory network differentiation [14, 15]. Once we have gene regulatory network estimates for each phenotype, we can model the problem of estimating the transition matrix within a regression framework. With this formulation, we solve for the *m*×*m* matrix that best describes the transformation between phenotypes (1). More specifically, MONSTER predicts the change in edge-weights for a transcription factor, indexed *i*, in a network based on all of the edge-weights in the baseline phenotype network.

$$E\left[b_{i}-a_{i}\right]=\tau_{1,i}a_{1}+\dots+\tau_{m,i}a_{m} $$

where *b*_*i*_ and *a*_*i*_ are column-vectors in **B** and **A** that describe the regulatory targeting of transcription factor *i* in the final and initial networks, respectively.

In the simplest case, this can be solved with normal equations,

$$\hat{\tau}_{i}=\left(A^{T}A\right)^{-1}A^{T}(b_{i}-a_{i}) $$

to generate each of the columns of the transition matrix **T** such that

$$\hat{\mathbf{T}}=\left[\hat{\tau}_{1},\hat{\tau}_{2},\dots,\hat{\tau}_{m}\right] $$

The regression is performed *m* times corresponding to each of the transcription factors in the data. In this sense, columns in the transition matrix can be loosely interpreted as the optimal linear combination of columns in the initial state adjacency matrix which predict the column in the final state adjacency matrix. The interpretation of the transition matrix can be best understood by comparing it to the identity matrix. A transcription factor, *i*, that does not alter its regulatory targets between states will have expected values of 0 for all entries in column *i*, with the exception of entry *i*. In the context of discovering changes in network configurations for a transcription factor, we are most interested in evaluating the degree to which each column has non-zero values for all non- *i* ^*th*^ entries. In essence, we are describing one network as a linear combination of another network. Numerous biological mechanisms, such as the formation of protein complexes, protein inactivation, post-translational modification, epigenetics, etc. allow for the systematic modification of network structures and drive the changes that are detected in the transition matrix (see Supporting Information).

This framework allows for the natural extension of constraints such as *L*1 and/or *L*2 regularization (see Supporting Information). For the analysis we present in this manuscript, we use the normal equations and do not impose a penalty on the regression coefficients. The inclusion or exclusion of this feature should depend primarily on assumptions of the underlying network mechanisms. L1 regularization, for example, will tend to infer a sparse transition matrix. This is reasonable in contexts in which the regulatory targeting pattern of TFs is not expected to change for the vast majority of TFs. For our four observational studies of COPD, a highly complex disease, it is not reasonable to assume that transcription factors differ in a sparse manner.

**Computing the transcription factor involvement:** For a transition between two nearly identical states, we expect that the transition matrix would approximate the identity matrix. However, as initial and final states diverge, there should be increasing differences in their corresponding gene regulatory networks and, consequently, the transition matrix will also increasingly diverge from the identity matrix. In this model, the transcription factors that most significantly alter their regulatory targets will have the greatest “off-diagonal mass” in the transition matrix, meaning that they will have very different targets between states and so are likely to be involved in the state transition process. We define the “differential transcription factor involvement” (dTFI) as the magnitude of the off-diagonal mass associated with each transcription factor, or,

2 $$ \hat{dTFI_{j}}=\frac{\sum_{i=1}^{m}I\left(i\ne j\right)\hat{\tau}_{i,j}^{2}}{\sum_{i=1}^{m}\hat{\tau}_{i,j}^{2}}  $$

where, $\hat {\tau _{i,j}}$ is the value of the element in the *i* ^*th*^ row and *j* ^*th*^ column in the transition matrix, corresponding to the *i* ^*th*^ and *j* ^*th*^ transcription factors. To estimate the significance of this statistic, we randomly permute sample labels *n*=400 times across phenotypes. From these 400 permuted results, we infer the standard error for each estimate under the null. *P*-values are determined by comparing the observed estimate to this standard error and FDR values is computed from these p-values (see Supporting Information).

## Results

### MONSTER finds significantly differentially involved transcription factors in COPD with strong concordance in independent data sets

As a demonstration of the power of MONSTER to identify driving factors in disease, we applied the method to case-control gene expression data sets from four independent Chronic Obstructive Pulmonary Disease (COPD) cohorts: Evaluation of COPD Longitudinally to Identify Predictive Surrogate Endpoints (ECLIPSE) [16, 17], COPDGene [18–20], Lung Genomics Research Consortium (LGRC) [21] and Lung Tissue from Channing Division of Network Medicine (LT-CDNM) [22]. The tissues assayed in ECLIPSE and COPDGene were whole blood and peripheral blood mononuclear cells (PBMCs), respectively, while homogenized lung tissue was sampled for LGRC and LT-CDNM.

As a baseline comparison metric, we evaluated the efficacy of applying commonly used network inference methods on these case-control studies. In analyzing phenotypic changes, networks are generally compared directly, with changes in the presence or weight of edges between key genes being of primary interest. It is therefore reasonable to assume that any reliable network results generated from a comparison of disease to controls will be reproducible in independent studies. We investigated whether this is the case for our four COPD data sets using three widely used network inference methods - Algorithm for the Reconstruction of Gene Regulatory Networks (ARACNE)[23], Context Likelihood of Relatedness (CLR)[24], and Weighted Gene Correlation Network Analysis (WGCNA) [25] - computing the difference in edge weights between cases and controls for each of the four studies. We found no meaningful correlation (*R* ^2^<.01) of edge weight difference across any of the studies regardless of network inference method or tissue type (Additional file 1: Supporting Figure S3). Edge weight differences, even when very large in one study, did not reproduce in other studies. This suggests that a simple direct comparison of edges between inferred networks is insufficient for extracting reproducible drivers of network state transitions. This finding may be unsurprising given the difficulty in inferring individual edges in the presence of heterogeneous phenotypic states, technical and biological noise with a limited number of samples.

**Fig. 2:**
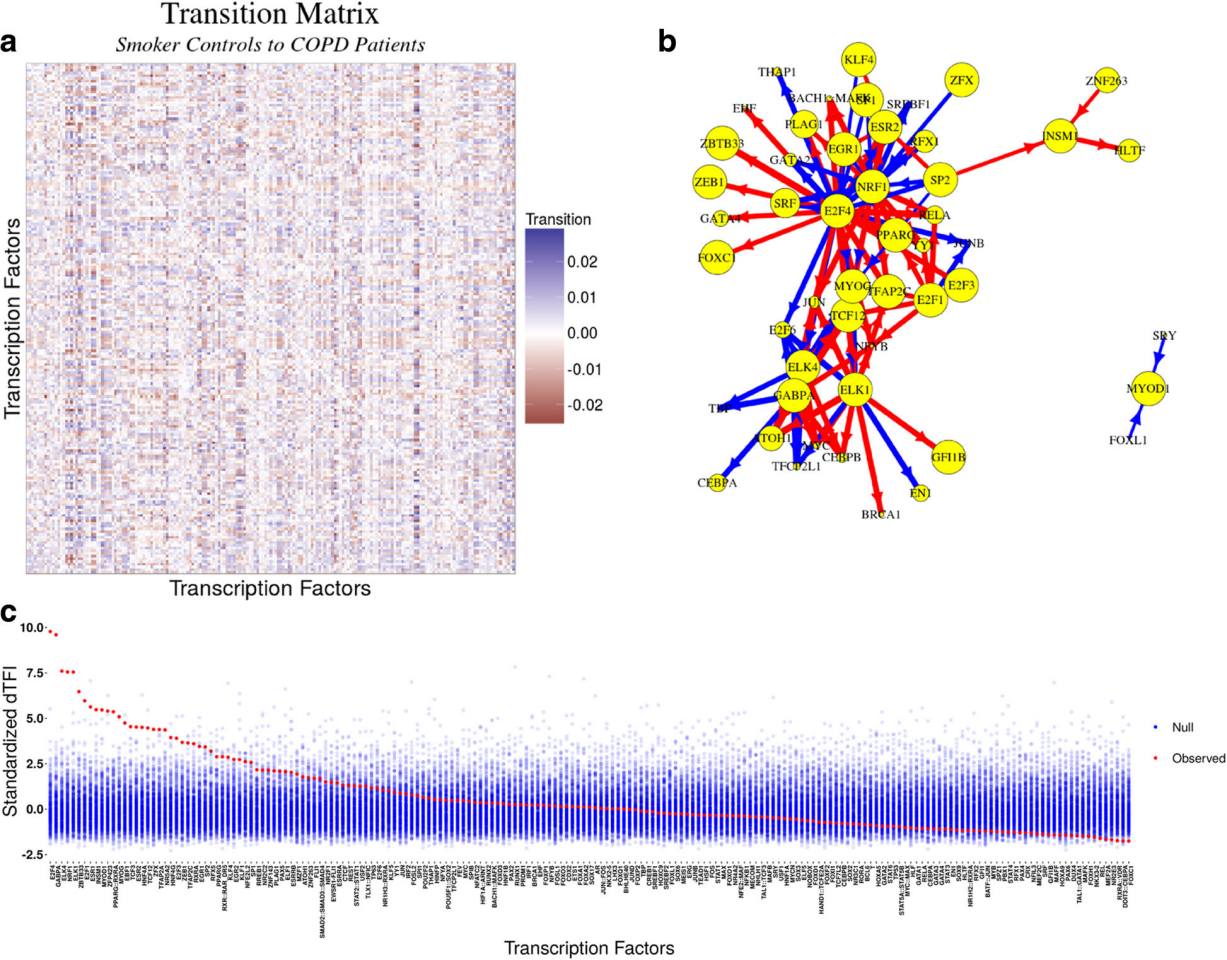
MONSTER analysis results in the ECLIPSE study. **a** Heatmap depicting the transition matrix calculated for smoker controls “transitioning” to COPD by applying MONSTER to ECLIPSE gene expression data. For the purposes of visualization, the magnitude of the diagonal is set to zero. **b** A network visualization of the 100 largest transitions identified based on the transition matrix in (**a**). Arrows indicate a change in edges from a transcription factor in the Smoker-Control network to resemble those of a transcription factor in the COPD network. Edge thickness represents the magnitude of the transition and node (TFs) sizes represent the dTFI for that TF. Blue edges represent a gain of targeting features and red represents the loss. **c** The dTFI score from MONSTER (red) and the background null distribution of dTFI values (blue) as estimated by 400 random sample permutations of the data

The lack of replication in edge-weight differences between independent data sets representing similar study designs indicates that we need to rethink how we evaluate network state transitions. MONSTER provides a unique approach for making that comparison. In each of the four COPD data sets, we used MONSTER to calculate the differential transcription factor involvement (*dTFI*, Eq. 2) for each transcription factor and used permutation analysis to estimate their significance (Fig. 2, Additional file 1: Additional Figures S1-S3). We observed strongly significant (*p*<1*e*−15) correlation in dTFI values for each pairwise combination of studies. In addition, out of the top 10 most differentially involved transcription factors in the ECLIPSE and COPDGene studies, we found 7 to be in common. Furthermore, three of these seven transcription factors (GABPA, ELK4, ELK1) also appeared as significant in the LGRC results with FDR <0.01 and each of the top five ECLIPSE results were among the top seven in the LT-CDNM results (Additional file 1: Additional Table S1, Additional file 1: Additional Figure S3). This agreement is quite striking considering that the there was almost no correlation in the edge-weight differences across these same studies when we tested the other methods. But it is exactly what we should expect—that the same method applied to independent studies of the same phenotypes should produce largely consistent results.

Many of the top dTFI transcription factors, especially those identified by MONSTER across all four studies, are biologically plausible candidates to be involved in the etiology of COPD (Additional file 1: Additional Table S1, Additional file 1: Additional Figures S1-S3). For example, E2F4 is a transcriptional repressor important in airway development [26] and studies have begun to demonstrate the relevance of developmental pathways in COPD pathogenesis [27].

Some of the greatest effect sizes across all four studies were found for SP1 and SP2. An additional member of the SP transcription factor family, SP3, has been shown to regulate HHIP, a known COPD susceptibility gene [28]. Both SP1 and SP2 form complexes with the E2F family [29, 30] and may play a key role in the alteration of E2F4 targeting behavior. Furthermore, E2F4 has been found to form a complex with EGR-1 (a highly significant transcription factor in ECLIPSE and LT-CDNM) in response smoke exposure, which may lead to autophagy, apoptosis and subsequently to development of emphysema [31].

Mitochondrial mechanisms have also been associated with COPD progression [32]. Two of most highly significant transcription factors based on dTFI in ECLIPSE were NRF1 and GABPA (FDR<.001). Indeed, these TFs had highly significant dTFI (FDR<0.1) in all four studies. NRF1 regulates the expression of nuclear encoded mitochondrial proteins [33]. GABPA, also known as human nuclear respiratory factor-2 subunit alpha, may have a similar role in nuclear control of mitochondrial gene expression. Furthermore, GABPA interacts with SP1 [34] providing evidence of a potentially shared regulatory mechanism with E2F4.

**Fig. 3:**
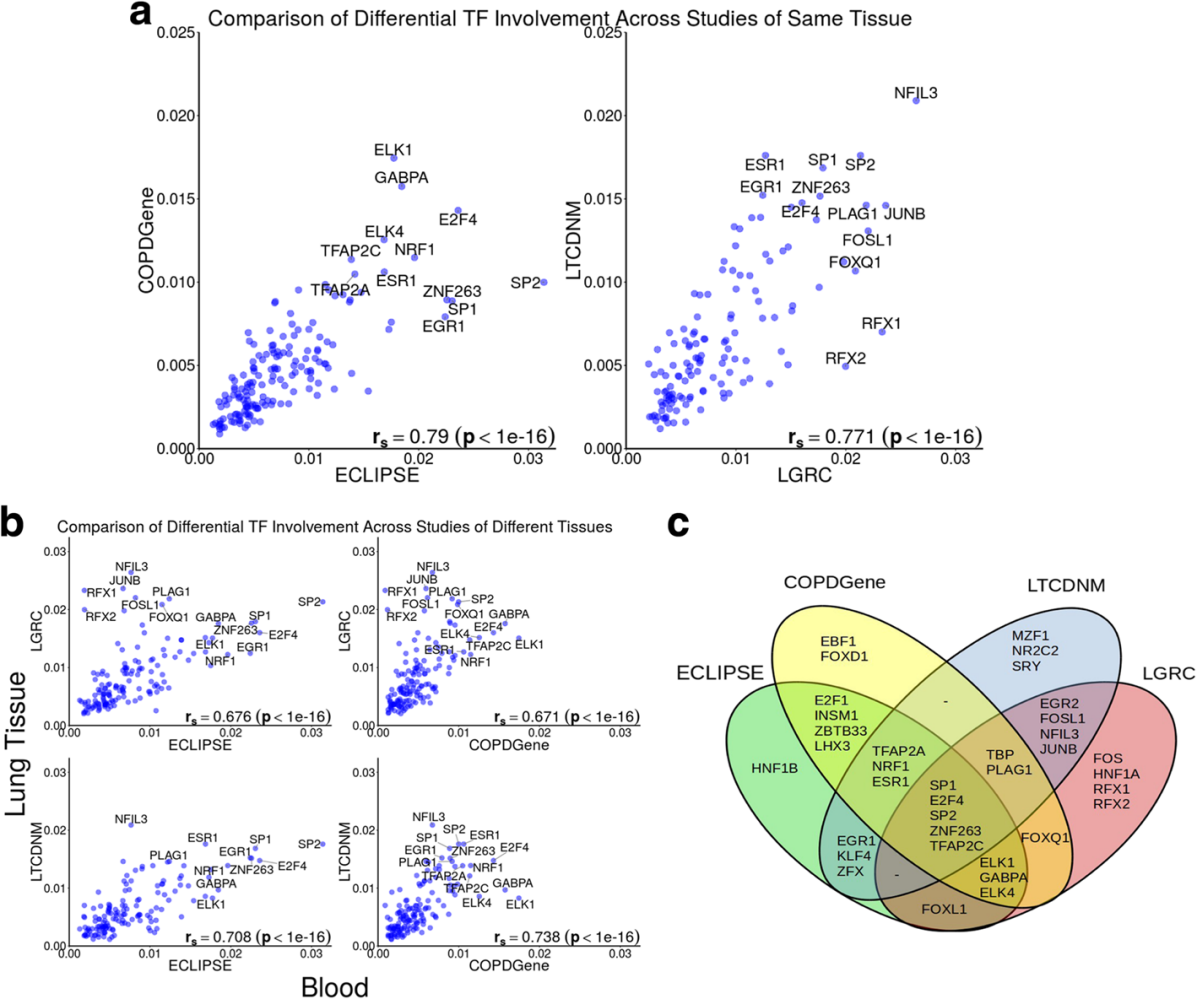
Strong reproducibility in top differential transcription factor involvement found in case-control COPD studies. ECLIPSE and COPDGene profiled gene expression in whole-blood and PBMC while the gene expression data in LGRC and LT-CDNM were assayed in lung tissue. **a** Results for studies with gene expression data obtained from the same-tissue. Both the blood based (left) and lung tissue studies (right) demonstrate very high Spearman correlation of differential involvement. **b** Despite using data from different sources we found agreement between studies of different tissues. **c** Venn diagram depicting the top 20 transcription factors found in each study. The union of all top 20 lists contains 36 transcription factors

Overall, we found a strong correlation across studies in transcription factors identified as significantly differentially involved (Fig. 3 a-b). It is reassuring that we find the strongest agreement when comparing studies that assayed similar tissues. However the fact that we see similar dTFI signal across studies involving different tissue types is also notable as it suggests that the transition from smoker control to disease phenotype affects multiple tissues and supports the growing evidence for a role in immune response in COPD pathogenesis.

Gene regulatory networks, and results derived from their comparison, are notoriously difficult to replicate across studies [35]. The four studies we used each has unique aspects, including the choice of microarray platform, study demographics, location, time, and tissue. Nevertheless, MONSTER identified similar sets of transcription factors associated with the transition between cases and controls. This consistency in biologically-relevant transcription factors, associated with the transition from the control phenotype to disease, in four independent studies suggests that MONSTER can provide not only robust network models, but also can identify reliable differences between networks.

Despite the overall consistency, some transcription factors had variable *dTFI* across studies. For example, using the LGRC dataset, we discovered a highly significant (*FDR*<.0001) differential targeting pattern involving the transcription factors RFX1 and RFX2 (Additional file 1: Additional Table S1). However, these same TFs were not identified as potential drivers of the control to COPD transition in either the ECLIPSE or COPDGene study. This difference is likely due the differences in tissue type as the RFX family transcription factors are known to regulate ciliogenesis [36]. Cilia are critical for clearing mucous from the airways of healthy individuals, but disruption can lead to infection and potentially to chronic airflow obstruction [37–39].

**Fig. 4:**
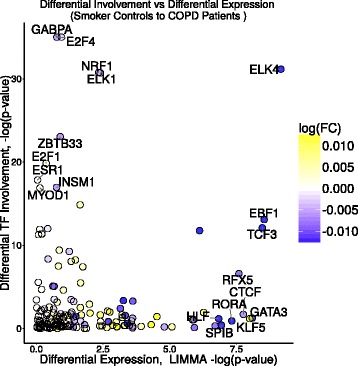
Differentially involved transcription factors are not necessarily differentially expressed. A plot of the differential expression versus the differential involvement for transcription factors based on our analysis of the ECLIPSE data. MONSTER commonly finds transcription factors which are differentially involved but are expressed at similar levels across cases and controls. Importantly, these transcription factors would not have been identified using conventional differential expression methods. This demonstrates the unique potential MONSTER has for discovery beyond standard gene expression analysis

The hypothesis behind MONSTER is that each phenotype has a unique gene regulatory network and that a change in phenotypic state is reflected in changes in transcription factor targeting. That hypothesis translates to an expectation that transcription factors driving change in phenotype will have the greatest *dTFI* scores. One might expect that these “driving transcription factors” would be also be differentially expressed. We compared *dTFI* to differential expression (ECLIPSE Fig. 4, other studies shown in Additional file 1: Additional Figure S4) and found that many of the transcription factors with high dTFI values were not differentially expressed. This suggests that there are other mechanisms, such as epigenetic modification of the genome or protein modifications, that alter the structure of the regulatory network by changing which genes are targeted by key transcription factors.

## Discussion

One of the fundamental problems in biology is modeling the transition between biological states such as that which occurs during development or as a healthy tissue transforms into a disease state. As our ability to generate large-scale, integrative multi-omic data sets has grown, there has been an increased interest in using those data to infer gene regulatory networks to model fundamental biological processes. There have been many network inference methods published, each of which uses a different approach to estimating the “strength” of interactions between genes (or between transcription factors and their targets). But all suffer from the same fundamental limitation: every method relies on estimating weights that represent the likelihood of an interaction between two genes to identify “real” (high confidence) edges. In comparing phenotypes, most methods then subtract discretized edges in one phenotype from those in the other to search for differences.

MONSTER represents a new way of looking at phenotypic transitions, but one that captures many aspects of what we should expect. First, we have to recognize that there is no single network that represents a phenotype, but that each phenotype is represented by a family of networks that all vary slightly from each other, yet which have essential features that are consistent with the phenotype. What this means is that each regulatory edge in a network representation has to be represented by continuous, rather than discrete, variables. This captures the fact that regulatory interactions are stronger in certain individuals and weaker in others, or present in some and absent in others, but that, on average, they represent a distribution.

Second, when we consider a change in phenotype, that will be reflected in altered patterns of gene expression, and ultimately in the networks that represent the phenotype. In a transition, some individuals will experience a greater change while others will experience a smaller change. But overall, regulatory patterns in the network will shift as the phenotype changes.

Third, the change in the gene regulatory network structure between phenotypes will be driven by changes in the connectivity of the regulators—the transcription factors that alter when, how, and how strongly genes are expressed. A natural hypothesis in this model is that the transition between phenotype is likely associated with the transcription factors that experience the greatest change in their regulatory patterns between states, and that the activation or inactivation of their target genes, and the functions carried out by those genes, likely reflect the phenotypic differences between states.

MONSTER captures these features, creating initial and final state network representations and estimating the change in transcription factor regulatory patterns by estimating a transition matrix. For each transcription factor, the “off diagonal mass” calculated as the differential transcription factor involvement (dTFI), identifies those transcription factors that are ultimately likely to drive the phenotypic state transition.

This approach has several limitations. For example, MONSTER does not attempt to infer specific regulatory mechanisms. Rather, by focusing on transcription factors, the goal of the method is to identify which transcription factors change their behavior between study groups. A deeper and more targeted investigation of the specific regulatory mechanisms that may be underlying these changes in transcription factor involvement would be needed to fully interpret the changes in targeting patterns that MONSTER is able to identify.

In applying MONSTER to four independent COPD gene expression data sets surveying both COPD and smoker controls, a highly consistent picture of the transcription factors associated with disease development emerges. This consistency is, to some, surprising as gene expression data is notoriously noisy, with each study finding sets of differentially expressed genes that often are not concordant. By focusing on transcriptional regulators, MONSTER seems to be able to separate a cleaner signal from the noise and one that makes some biological sense. Indeed, when one looks at the transcription factors found by MONSTER as associated with the transition, all are biologically plausible candidates which provide new and important opportunities for future molecular studies of COPD pathogenesis. It is also noteworthy that many of these transcription factors could not have been found through a simple differential expression analysis as their transcriptional levels do not change significantly between disease and control populations. Rather, it is the regulatory patterns of these transcription factors, possibly driven by epigenetic or other changes, that shifts with the phenotype.

## Conclusion

The systems biology research community has long framed the discussion of the transitions between phenotypes in terms of gene expression state space, in which a change in phenotype corresponds to a transition between one characteristic expression profile to another. Here we extend that framework to the gene regulatory network state space, recognizing that patterns of gene expression are driven by alterations in patterns of gene regulation—and therefore, changes in the corresponding gene regulatory network. This shift allows us to ask how phenotypic alterations, and the corresponding regulatory changes, are effected through transcription factor “rewiring,” and to identify those transcription factors that are altering their regulatory targets. Thus MONSTER represents a novel approach to identifying and understanding the factors that drive changes in biological states and one with broad potential for application in a range of systems.

## Additional file

Additional file 1 (1) A detailed description of the data used for the COPD network inference and analysis presented in the main text. (2) A detailed description of the MONSTER approach for defining network state transitions. (3) Various evaluations of the MONSTER method. (4) An illustration of the irreproducibility of network differences outside of the transition matrix formalism. (PDF 5417 kb)
