# Supplementary material for: Estimating drivers of cell state transitions using gene regulatory network models
Source: BMC Syst Biol. 2017 Dec 13;11:139. doi: 10.1186/s12918-017-0517-y (PMC5729420; doi:10.1186/s12918-017-0517-y)
Supplement: Additional file 1 — (1) A detailed description of the data used for the COPD network inference and analysis presented in the main text. (2) A detailed description of the MONSTER approach for defining network state transitions. (3) Various evaluations of the MONSTER method. (4) An illustration of the irreproducibility of network differences outside of the transition matrix formalism. (PDF 5417 kb) [file 12918_2017_517_MOESM1_ESM.pdf]

Overview of Supporting Information

This Supporting Information document is broken into four main sections that include:

- A description of the data used for the COPD network inference and analysis presented in the main text
- A detailed description of the MONSTER approach for defining network state transitions
- Various evaluations of the MONSTER method
- An illustration of the irreproducibility of network differences outside of the transition matrix formalism

Data for COPD Network Inference and Analysis

Sequence binding motifs

A regulatory network prior between transcription factors and target genes was created by using position weight matrices for 205 transcription factor motifs obtained from JASPAR 2014 (http://jaspar2014.genereg.net/), [21] and running Haystack[22] to scan the hg19 genome for occurrences of these motifs. Sequences were identified as hits for a transcription factor if they satisfied the significance threshold of $p < 10^{-5}$. We then used HOMER (http://homer.salk.edu/homer/ngs/index.html) [12] to identify transcription factor binding motifs that map to a window ranging from 750 base pairs downstream to 250 base pairs upstream of each gene's transcription start site under the assumption that transcription factors falling in this region may actively regulate expression of the gene.

ECLIPSE

Gene expression data from the ECLIPSE study (GSE54837) [27] was collected using blood samples from 226 subjects classified as non-smokers (6), smoker controls (84) or COPD (136). Blood samples from each individual were profiled using Affymetrix Human Genome U133 Plus 2.0 microarrays. CEL data files from these assays were RMA-normalized[13] in R using the Bioconductor package 'affy'[8]. Array probes were collapsed to 19,765 Entrez-gene IDs using a custom CDF[3] and the 220 samples for COPD or smoker control subjects were retained for analysis. Finally, genes were associated with potential regulatory transcription factors using a motif scan (described above). 1,553 genes were not associated with any transcription factor and excluded from further analysis, leaving 17,342 genes that were used to construct network models.

COPDGene

Gene expression data from the COPDGene study (GSE42057) [2, 26] was collected from blood samples obtained from 136 subjects classified as smoker controls (42) or COPD (94) and profiled on Affymetrix Human Genome U133 Plus 2.0 microarrays. Similar to the ECLIPSE data, CEL data files from these microarray assays were RMA-normalized using the 'affy' package and array probes were collapsed to Entrez-gene IDs using a custom CDF[3], yielding 18,960 genes. After removal of genes that did not match with our motif scan, the COPDGene data contained 17,253 genes.

LGRC

Gene expression data from 581 lung tissue samples in the LGRC (GSE47460) [1] was profiled using two array platforms: Agilent-014850 Whole Human Genome Microarray 4x44K G4112F and Agilent-028004 SurePrint G3 Human GE 8x60K arrays. LIMMA was used to background correct and normalize gene expression across samples within each of these two platforms. Genes that were represented by more than one probe were then removed and the expression data was merged between the two array platforms by matching probes that represented the same gene, leaving 17,573 genes. Next, batch effect due to the array platform was addressed by running ComBat [14]. Genes not present in our motif scan were then removed, yielding 14,721 genes. After normalization we filtered the samples included in the LGRC dataset by removing those that corresponded to subjects that (1) were not designated as either a COPD case or control (mostly subjects with Interstitial Lung Disease), (2) had a diagnosis of COPD, but spirometric

measures in the normal range, (3) had been identified as non-Caucasian, (4) had been labeled as a former smoker, but had zero or unknown pack years, (5) had high pre-bronchodilator FEV1/FVC ratios, or (6) had been taken as a biological replicate of another sample which was included. After removal of those samples we were left with 164 COPD cases and 64 controls for which we had gene expression data.

LTCDNM

Gene expression data from the LTCDNM (GSE76925)[23] was collected using HumanHT-12 BeadChips. Quality control was performed using quantile, signal-to-noise, correlation matrix, MA, and principal component analysis (PCA) plots using R statistical software (v 3.2.0) to identify outliers and samples with questionable or low-quality levels, distributions, or associations. This process yielded 151 samples for analysis, including 115 subjects classified as either diagnosed with COPD (87) or as a smoker control (28). After filtering for low variance and percentage of high detection p-values, 32,831 probes representing 20,794 genes were retained. The R package lumi [4] was then used for background correction, log2 transformation and quantile normalization. Finally, we collapsed probes to gene symbols based on maximum gene expression and removed genes that were not matched with our motif scan, yielding 14,273 genes.

TFs included in analysis

For each study, we identified transcription factors for which we had gene expression data, removing those transcription factors that lacked expression values. This mapping and filtering left 164 transcription factors in ECLIPSE and COPDGene, 148 in LGRC, and 145 in LTCDNM. MONSTER was run separately on each of these studies. Comparisons of differential transcription factor involvement across studies were performed using the 143 transcription factors that were common to all four studies.

MONSTER: MOdeling Network State Transitions from Expression and Regulatory data

The MONSTER algorithm conceptually consists of three parts: (1) inferring a gene regulatory network, (2) computing a transition matrix, and (3) quantifying the differential transcription factor involvement. We review each of these steps separately below.

Inferring Gene Regulatory Networks

In 2013, we described PANDA [9], a method for estimating gene regulatory networks that uses "message passing" [7] to integrate multiple types of genomic data. PANDA begins with a prior regulatory network based on mapping transcription factor motifs to a reference genome and then integrates other sources of data, such as protein-protein interaction and gene expression, to estimate a collective network. While PANDA has proven to be very useful in a number of applications [17, 11, 10], its iterative approach to edge weight optimization limits its utility in situations requiring a large number of network bootstrap estimations, including applications where the sample size is large [29].

To overcome this limitation in MONSTER we developed a regression-based approach that considers the available evidence of a gene regulatory "edge" in the network for each possible transcription factor-gene pair. This evidence can be divided into two components, referred to here as direct and indirect. Consider the edge between a gene that codes for a transcription factor, TF_i, and another gene. The direct evidence, $d_{i,j}$, can be estimated by the squared conditional correlation:

$$\hat{d}_{i,j} = cor\left(g_i, g_j | \{g_k : k \neq i, k \in \mathbf{TF_j}\}\right)^2,$$

where g_i is the gene which encodes TF_i, g_j is any other gene in the genome, and $\mathbf{TF_j}$ is the set of gene indices corresponding to known transcription factors with binding site in the promoter region of g_j. The correlation is conditioned on the expression of all other potential regulators of g_j based on the transcription factor motifs associated with g_j.

Naturally, the use of direct evidence alone inadequately captures regulatory relationships, which can be difficult to estimate due to systematic and technical noise as well as biological factors, such as transient protein-protein interactions and post-translational modifications, that may mask or modify a true regulatory effect. Therefore we want to complement our estimate of the likelihood of a regulatory

mechanism by aggregating the information from the gene expression patterns of all suspected targets of any given transcription factor.

PANDA achieves its superior performance in part by convergence towards an "agreement" across multiple sources of evidence, in essence requiring that large collections of gene expression patterns must agree with the proposed regulatory structure in order to claim an interaction. In MONSTER, we look for agreement between the gene expression patterns of large sets of co-targeted genes. We refer to this as "indirect evidence" and estimate this by once again using the regulatory prior. Here, we no longer consider transcription factors to be members of the set of genes and instead consider each of the m transcription factors to be binary classifications across the entire gene list. Class labels are determined by the presence or absence of a sequence binding motif for a given transcription factor in the promoter region of a gene. For each transcription factor, we use the gene expression patterns of all targeted genes against all non-targeted genes to build a classifier. In this manner we are assigning a higher score for edges connecting each transcription factor to genes which demonstrate an expression pattern more similar to the suspected targets.

Based on this, the indirect evidence between the two nodes, $\theta_{i,j}$, is estimated by the fitted probability that g_j belongs to the class of genes targeted by TF_i. We use a logistic regression on the gene expression data with outcome taken to be the existence or non-existence of a known sequence motif for TF_i in the promoter region of g_j.

$$logit\left(E\left[M_i\right]\right) = \beta_{0,i} + \beta_{1,i} g^{(1)} + \cdots + \beta_{N,i} g^{(N)}$$

where the response M_i is a binary vector of length p indicating the of the presence of a sequence motif for transcription factor i in the vicinity of each of the p genes. And where $g^{(k)}$ is a vector of length p representing the expression of genes in sample k.

For a given transcription factor-gene pair, the fitted values for each $TF_i - g_j$ pair define the "indirect" evidence $\theta_{i,j}$, which can be estimated by:

$$\hat{\theta}_{i,j} = \frac{e^{\hat{\beta}_{0,i} + \hat{\beta}_{1,i} g_j^{(1)} + \cdots + \hat{\beta}_{N,i} g_j^{(N)}}}{1 + e^{\hat{\beta}_{0,i} + \hat{\beta}_{1,i} g_j^{(1)} + \cdots + \hat{\beta}_{N,i} g_j^{(N)}}}$$

where $g_j^{(k)}$ is the measured gene expression for sample k at gene j.

We score each gene according to the strength of indirect evidence for a regulatory response to each of the transcription factors and combine this with the direct evidence of regulation. Combining our measures of direct and indirect evidence presents some challenges. Though both are bounded by [0,1] their interpretations are quite different. The direct evidence can be considered in terms of its conditional gene expression R^2 between nodes, while the indirect evidence is interpreted as an estimated probability. Therefore, we use a non-parametric approach to combine evidence. Specifically, the targets of each transcription factor are ranked and combined as a weighted sum, $w_{i,j} = (1-\alpha)\left[rank\left(\hat{d}_{i,j}\right)\right] + \alpha\left[rank\left(\hat{\theta}_{i,j}\right)\right]$, where α is a constant bounded between $[0, 1]$. Our choice of the weight is by default $\alpha = 0.5$, corresponding to an equal contribution of direct and indirect evidence. This parameter could be adjusted if the context of a study involved reason to prefer one source of evidence over the other.

Computation of MONSTER's transition matrix

The hypothesis behind MONSTER is that different phenotypes are characterized by distinct regulatory networks and that transitions between networks are associated with large-scale changes in the regulatory structure of the network. Essentially, transcription factors gain or lose targets and in doing so, alter the structure of the network from one phenotypic state to another. The task of identifying meaningful network transitions then becomes an evaluation of the relative refinement of edge weights.

Our analysis of validation data sets (shown below) indicates that the reconstructed networks are strongly driven by the structure of the motif prior, with small changes defining differences between phenotypes. Hence, in comparing networks between phenotypes, the problem becomes one of of understanding changes in edges that have relatively low signal and high noise. In other words, state transitions are characterized by a large number of individually unreliable edge weights.

Consider two adjacency matrices, $\mathbf{A}$ and $\mathbf{B}$, that represent two gene regulatory networks estimated from a case-control study. Each matrix has dimensions $(p \times m)$ representing the set of p genes targeted by m transcription factors. We seek a matrix, $\mathbf{T}$, such that

$$\mathbf{B} = \mathbf{AT} + \mathbf{E}$$

where $\mathbf{E}$ is our error matrix, which we want to minimize. Intuitively, we may frame this as a set of m independent regression problems, where m is the number of transcription factors and also the column rank of $\mathbf{A}$, $\mathbf{B}$, $\mathbf{T}$, and $\mathbf{E}$. For a column in $\mathbf{B}$, $\mathbf{b}_i$, we note that a corresponding column in $\mathbf{T}$, τ_i, represents the ordinary least squares solution to

$$E\left[\mathbf{b}_i\right] = \tau_{i1}\mathbf{a}_{1i} + \tau_{i2}\mathbf{a}_{2i} + \cdots + \tau_{im}\mathbf{a}_{mi}$$

or alternatively expressed

$$\begin{bmatrix} \mathbf{b}_{i1} \\ \mathbf{b}_{i2} \\ \vdots \\ \mathbf{b}_{ip} \end{bmatrix} = \tau_{1,i} \begin{bmatrix} \mathbf{a}_{11} \\ \mathbf{a}_{21} \\ \vdots \\ \mathbf{a}_{p1} \end{bmatrix} + \cdots + \tau_{p,i} \begin{bmatrix} \mathbf{a}_{1p} \\ \mathbf{a}_{2p} \\ \vdots \\ \mathbf{a}_{pp} \end{bmatrix} + \begin{bmatrix} \epsilon_{i1} \\ \epsilon_{i2} \\ \vdots \\ \epsilon_{ip} \end{bmatrix}$$

where $E\left[\epsilon_{ij}\right] = 0$. This can be solved with normal equations,

$$\begin{aligned} \tau_i &= \left(\mathbf{A}^T\mathbf{A}\right)^{-1}\mathbf{A}^T\mathbf{b}_i \\ \mathbf{T} &= \left[\tau_1, \tau_2, \ldots, \tau_m\right] \end{aligned}$$

which produces the least squares estimate. In other words, the loss function $L\left(\mathbf{T}\right) = \sum_{gene=1}^{N} ||\mathbf{B}_{gene} - \mathbf{A}_{gene}\mathbf{T}||^2$ is minimized.

It is easy to see how this allows for a straightforward extension via the inclusion of a penalty term. For example, an L_1 regularization[28] can be used to create an identity penalty model matrix for each column regression such that only the kth diagonal element is 0 and all other diagonals are 1. This gives unpenalized priority for the kth regression coefficient in the kth regression model:

$$\mathbf{Q}_{i,j} = \begin{cases} 1 & for\, i = j \neq k \\ 0 & elsewhere \end{cases},$$

which results in the minimization of the penalized residual sum of squares

$$PRSS\left(\mathbf{T}_{\cdot,\boldsymbol{k}}\right) = \sum_{i=1}^{p}\left(\mathbf{B}_{i,k} - \sum_{j=1}^{m} A_{i,j}\mathbf{T}_{j,k}\right)^2 + \lambda\sqrt{\mathbf{T}'_{\cdot,k}\boldsymbol{Q}\mathbf{T}_{\cdot,k}}$$

Although not used in the analysis presented in the main text, an implementation of this extension is available in the R package MONSTER.

Analyzing the Transition Matrix

The derivation described above illustrates a key feature of the MONSTER method. Specifically, that the transition matrix ($\mathbf{T}$) reduces the case-control network transformation from a set of $2 \times p \times m$ estimates to a set of $m \times m$ estimates that are more easily interpreted. We can think of a column, τ_i, on the matrix $\mathbf{T}$ as containing the linear combination of regulatory targets of TF_i in $\mathbf{A}$ that best approximates the regulatory targets of TF_i in $\mathbf{B}$. As one would expect, a large proportion of the matrix "mass" would be on the diagonal for those transcription factors which do not change regulatory behavior between case and control. It is therefore of interest to evaluate values off of the diagonal as indicative of a network transition.

There are many biological processes involved in gene regulation that may differ between phenotypic states, including RNA degradation, post-translational modification, protein-level interactions and epigenetic alterations. These all have the ability to impact transcription factor targeting without impacting the expression level of the transcription factor itself. Because our hypothesis is that changes in phenotype are associated with changes in regulatory networks, we want to identify those transcription factors that have undergone significant overall changes in behavior between states. As a measure to quantify such changes, we define the differential Transcription Factor Involvement (dTFI),

$$s_j = \frac{\sum_{i=1}^{m} I\left(i \neq j\right)\tau_{i,j}^2}{\sum_{i=1}^{m}\tau_{i,j}^2}.$$

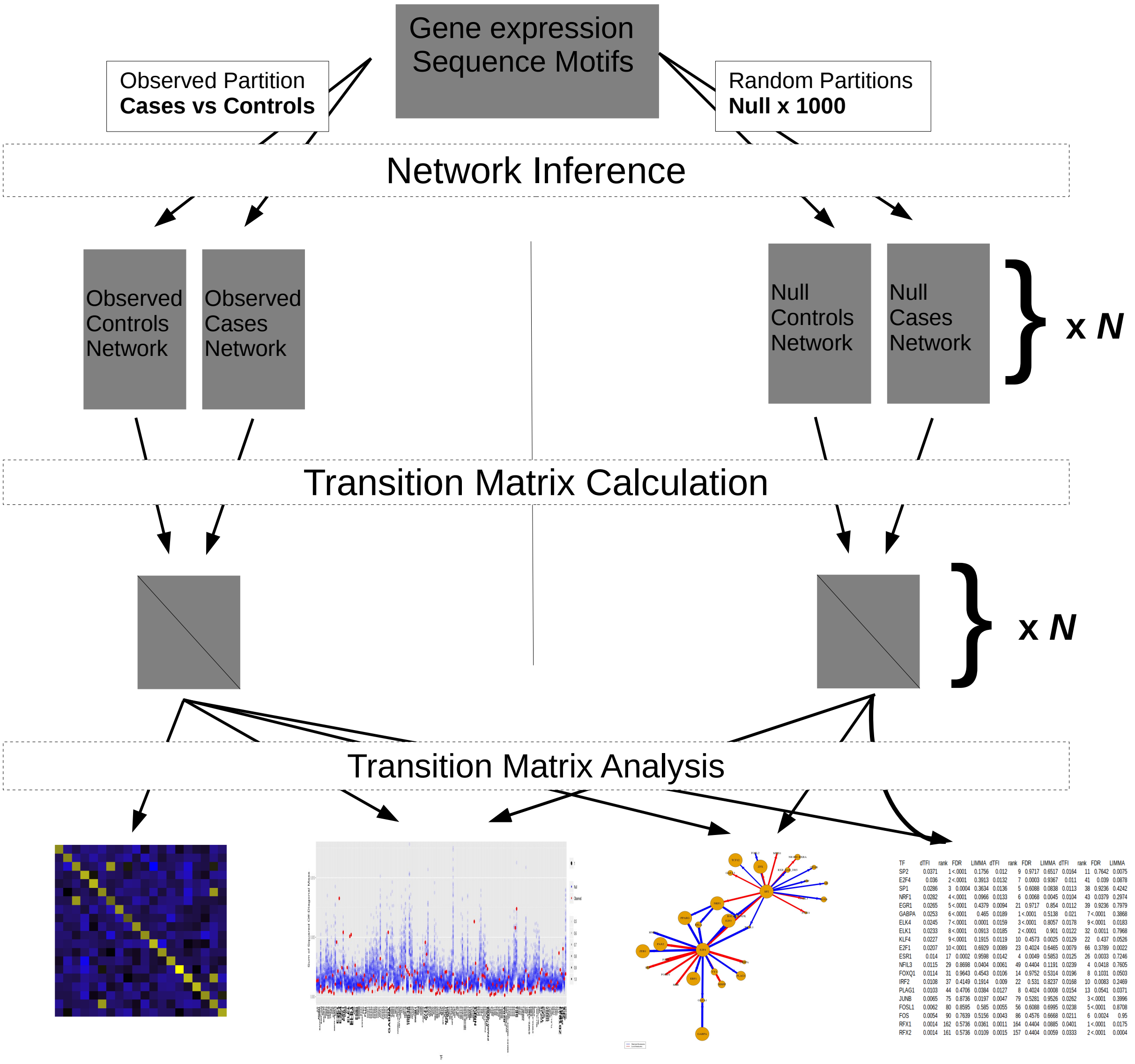

Supporting Figure 1: **Overview of MONSTER analysis workflow.** (1) Network inference is computed separately to subsets of the gene expression data including the case group, the control group and N permutations of the case and control labels. (2) The transition matrix is estimated between the cases and controls and each of the pairs of permuted "case" and "control" groups. (3) The transition matrix computed between the case and control group is interpreted within the context of the N matrices estimated for the permuted groups.

The dTFI can be loosely interpreted as the proportion of transcription factor targeting that is gained from or lost to other available transcription factors as the state changes. It is a statistic on the interval $[0, 1]$ that can be used to identify transitions which are systematic, informative, and non-arbitrary in nature. In other words, the dTFI can capture edge weight signal for which there is an attributable regulatory pattern based on the inferred networks.

The distribution of the dTFI statistic under the null has a mean and standard deviation that depends to a large extent on the motif-based network prior structure. In particular, we find that both mean and standard deviation of the dTFI are higher for transcription factors that have fewer prior regulatory targets. From a statistical perspective, transcription factors with relatively more targets are able to generate more stable targeted expression patterns, which leads to more consistent estimates in "agreement". From a biological perspective, increased motif presence may indicate that transcription factors are more likely to be involved in "housekeeping" or tissue specific processes that are unlikely to change between cases and controls.

We address the dependence of the null distribution of the dTFI on the motif structure using the following resampling procedure (Supporting Figure 1):

0. Gene regulatory networks are reconstructed based on a prior regulatory structure and gene expression from case and control samples and the transition matrix and the dTFI values for each transcription factor are computed.

1. Gene expression samples are randomly assigned as case and control forming null-case and null-control groups with sizes reflecting the true case and control groups.

2. Gene regulatory networks are reconstructed for the null-case and null-control groups with the same prior regulatory structure.

3. The transition matrix algorithm is applied to the two null networks.

4. The dTFI is calculated for each transcription factor based on the computed null transition matrix.

5. Steps 1-4 are repeated n times.

For the analysis presented in the main text, we set $n = 400$. This procedure allows us to estimate a background distribution of dTFI values based on the underlying motif prior network structure and therefore test the significance of observed dTFI values between cases and controls.

Validation of the MONSTER Approach

MONSTER recovers network edges in *in silico*, *Escherichia coli* and Yeast (*Saccharomyces cerevisiae*)

For its initial step, MONSTER uses gene expression together with a prior network structure to infer regulatory network edges. For method testing and validation of MONSTER's network estimates we used four data sets of increasing biological complexity: (1) *in silico*, (2) *Escherichia coli*, and (3) *Saccharomyces cerevisiae* (yeast) expression data together with simulated motif priors derived from reference networks and (4) yeast expression data together with a biological motif prior generated independently of the reference. For data set (4), we used the yeast motif prior, 106 gene expression samples from transcription factor knockout or overexpression conditions, and ChIP gold standard described in Glass *et. al.*[9]. Data for the first three sources was obtained from the 2012 DREAM5 challenge data set[19]. This challenge asked contestants to infer gene networks from expression data alone, using a reference standard for evaluation. For the purposes of validating MONSTER, we instead started with the reference network and randomly perturbed TF-gene pairs to create the type I and type II error rates consistent with biological yeast motif prior used in the fourth data set. Specifically, if an edge appeared in the reference network, that edge appeared in the simulated motif data with probability 0.3; if an edge was absent from the reference network, that edge appeared in the simulated motif data with probability 0.1. These probabilities result in an area under the Receiver-Operator Characteristic curve (AUC-ROC) of approximately 0.7 for prediction of the reference edges by the simulated edges.

For each of the data sets, we evaluated the accuracy of MONSTER's network inference method using AUC-ROC. For the DREAM5 data sets we applied MONSTER to the expression data together with the simulated priors and used the original reference networks as our gold-standards. For the fourth

AUC-ROC for edge weight differences vs Transition Matrix using various NI methods

NI Method	Network AUC	edge weight differences	MONSTER
Pearson	.704	.512 (p=.61)	.688 (p<.0001)
TOM	.703	.51 (p=.62)	.689 (p<.0001)
ARACNE	.515	.523 (p=.58)	.566 (p=.09)
CLR	.694	.57 (p=.19)	.814 (p<.0001)

Supporting Table 1: **Comparison of edge weight difference to Transition Matrix in simulated case-control gene expression**. Several network inference methods were run on our *in silico* case-control data. The overall network area under the curve of the receiver-operator characteristic (AUC-ROC) was performed for each method averaged across cases and controls. The naive transcription factor-transcription factor transitions were calculated as the difference in transcription factor-transcription factor edge weight between cases and controls. The transition matrix transcription factor-transcription factor transitions used the absolute transition matrix values.

data set we applied MONSTER to the expression and motif data, and used the ChIP-chip data as our gold-standard. We found that in all four of these data sets, the accuracy of the estimated edges from MONSTER's network inference was superior to the accuracy of the input motif prior data (Supporting Figure 2).

MONSTER accurately predicts transcription factor transitions in *in silico* gene expression data

We next used simulated data to evaluate MONSTER's transition matrix. To begin, we randomly generated a "true" control adjacency matrix, $\mathbf{M}_0$, which contained information for all possible edges between $m = 100$ transcription factors and $p = 10,000$ genes with "edge weights" sampled from a standard uniform distribution. We then defined a state transition matrix, $\mathbf{T}$, with diagonal elements set equal to one and $1,000$ random off-diagonal elements (representing random pairs of transcription factors) set equal to values sampled from a uniform random distribution between -1.0 and 1.0. These off-diagonal elements (transcription factor pairs) ultimately represent the transitions that we seek to recover and their corresponding values represent the magnitude of the regulatory transition. Finally, based on $\mathbf{M}_0$ and $\mathbf{T}$ we defined the "true" cases network as $\mathbf{M}_1 = \mathbf{T}\mathbf{M}_0$.

Next, we generated two *in silico* gene expression datasets, one each for the case and control networks. To do this, we sampled 500 times from each of two multivariate Gaussian distributions with the variance-covariance matrix, Σ, defined as $\mathbf{M}_0\mathbf{M}_0'$ and $\mathbf{M}_1\mathbf{M}_1'$ for controls and cases, respectively. We note that we scaled the magnitude of the diagonal elements of Σ by 4 to simulate noise in the *in silico* data. This value was chosen such that the networks predicted using the *in silico* gene expression data had an AUC-ROC of approximately .70 when evaluated using the "true" networks (see below).

We next used this simulated data to reconstruct networks using several commonly used network inference methods, including the Pearson correlation (used in WGCNA) [15] [16], Topological Overlap Measure (TOM) [25], Algorithm for the Reconstruction of Gene Regulatory Networks (ARACNE) [20], and Context Likelihood of Relatedness (CLR) [5]. The implementation of each method was from the R package nettools [6].

We next constructed a gold-standard for our network transitions, defined as $\mathbf{T}_{GS} = ceil(|\mathbf{M}|)$. For each of the five network inference methods, we then evaluated the accuracy of two potential approaches for identifying network alterations. First, we simply subtracted edge weights between the inferred cases network and the inferred controls network and selected those edges that extended between the 100 TFs in our model (excluding those genes that were not TFs). Second, we used MONSTER to predict the transition needed to map the control network to the case network. The results are summarized in Supporting Table 1. For each of the network inference methods tested, we found that the transition matrix showed substantial improvement over the edge weight difference method in identifying transitions between transcription factors. In all cases, the edge weight difference (column 3) was not statistically significant for predicting transitions, but when the transition matrix was used (column 4) a strong predictive signal appeared.

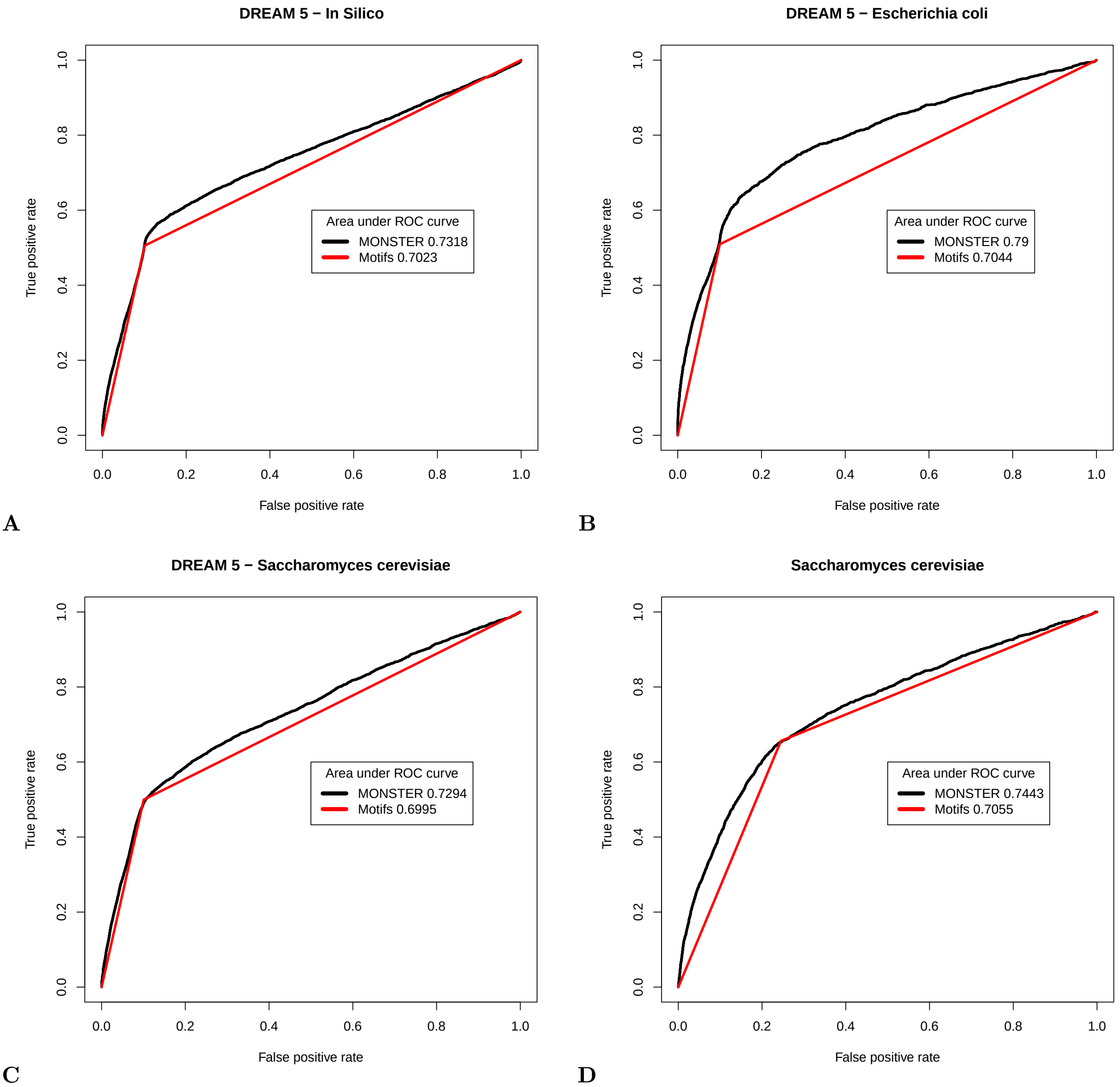

Supporting Figure 2: Receiver-Operator Characteristic curves for three DREAM 5 data sets (A) *in silico*, (B) *Escherichia coli*, (C) *Saccharomyces cerevisiae*, and an (D) additional *Saccharomyces cerevisiae* data set as described in Glass *et. al.*[9]. The prior network for each of the DREAM5 data set analyses was derived from the validation standard, with error introduced (both type I and type II) bringing the area under the ROC curve to ≈ 0.70. In the other *Saccharomyces cerevisiae* data set analysis, sequence motifs were used as the prior and a ChIP-chip derived network was used as the validation standard. In each of these tests, we observed a measurable improvement in performance of MONSTER's network inference method over the prior.

MONSTER finds significant protein-protein interactions

There are numerous biological regulatory mechanisms that may play a role in transitions between phenotypic states. Of particular interest to us are those that are not readily detectable via conventional methods for the analysis of gene expression data. For example, gene regulation involves complex processes in which transcription factors, either singly or in multiprotein complexes, bind to DNA in the region of a gene to activate or repress the transcriptional process. Such multi-protein interactions create combinatorial complexity that can explain much of the variation in organism complexity which is unexplained by gene expression alone [18].

As reported in the main text, we ran MONSTER on data from 84 smoker controls and 136 COPD subjects in the ECLIPSE study. To test whether MONSTER could reliably detect protein-protein interactions between regulatory transcription factors, we evaluated whether our estimated transitions between case and control COPD networks in this analysis recapitulated known protein-protein interactions, as reported in Ravasi *et. al.*[24] and processed in Glass *et. al.*[11]. This dataset contained 223 interactions between the transcription factors we used as input of our model; of these, 39 were self-interacting and were removed. We attempted to predict the remaining 184 interactions between transcription factors using MONSTER.

We used the absolute value of the transition matrix and tested whether that value predicted protein-protein interactions based on the area under the ROC curve. To assess the significance of AUC-ROC, we also applied this evaluation to the 400 "random" transition matrices generated based on the randomized phenotypic labels. MONSTER achieved an AUC-ROC score of .548, suggesting predictive power to identify known PPI between transcription factors. While weak, this result exceeded all randomized phenotype results and was significant at $p < .0025$. This indicates that MONSTER is able to extract a small but significant protein interaction signal from highly obfuscated data.

Irreproducibility of network inference methods in estimating transcription factor - gene edge-weights in COPD

Conceptually, MONSTER is comprised of two elements. The first infers gene regulatory networks from transcriptional data while the second uses the networks inferred for two different phenotypes to calculate the transition matrix between states. Instead of using the second part of the MONSTER approach to understand the transition between one state and another, one could imagine instead substracting the edge-weights predicted for two networks and using those differences to define a transition between two phenotypic states. To test whether this is a reasonable approach we examined the reproducibility of edge weight differences between case and control networks estimated for four COPD datasets using MONSTER's network reconstruction approach as well as three other widely used network inference methods: Algorithm for the Reconstruction of Gene Regulatory Networks (ARACNE), Context Likelihood of Relatedness (CLR), and the standard Pearson correlation used in such methods as Weighted Gene Correlation Network Analysis (WGCNA).

We used each of the four methods to separately estimate networks for cases and controls in each of the COPD studies. We then calculated the difference between case and control edges (differential edge weights) in each study for each method. We reasoned that if edge-differences were reflective of biologically meaningful associations, these should be present in each study and should appear as a correlated set of differential edge weights.

We plotted the differential edge weights for each pairwise combination of studies (Supporting Figure 3) and found that the differential edges found by ARACNE, CLR, WGCNA and MONSTER were almost entirely study specific, meaning that edges are found in one study comparing smoker controls to COPD patients are not found in a second study comparing the same phenotypes. Clearly, evaluation of individual edge-weight differences is not a reproducible approach for comparing inferred networks and stands in stark contrast to the highly reproducible set of differentially-involved set of transcription factors that we were able to identify across all four studies (as presented in the main text).

References

[1] Lung genomics research consortium (lgrc). 2015. Accessed: 2016-02-02.

A Top significantly differentially involved transcription factors

	ECLIPSE			COPDGene			LGRC			LTCDNM		
transcription factor	dTFI	rank	FDR	dTFI	rank	FDR	dTFI	rank	FDR	dTFI	rank	FDR
SP2	.0314	1	.0357	.0100	9	.6812	.0213	6	.3752	.0176	2	.7438
E2F4	.0236	2	<.0001	.0143	3	<.0001	.0160	14	.037	.0148	7	<.0001
SP1	.0230	3	.1551	.0089	18	.7721	.0179	10	.3594	.0169	4	.5516
ZNF263	.0226	4	.311	.0089	16	.3372	.0177	11	.7716	.0152	6	.927
EGR1	.0224	5	.1242	.0079	23	.7597	.0124	28	.6892	.0152	5	.5305
NRF1	.0196	6	<.0001	.0115	5	.0304	.0122	30	<.0001	.0139	11	.0558
GABPA	.0185	7	<.0001	.0157	2	<.0001	.0176	12	<.0001	.0097	32	.0853
ELK1	.0177	8	<.0001	.0174	1	<.0001	.0151	17	<.0001	.0083	40	.2099
ZFX	.0175	9	<.0001	.0076	24	.8366	.0103	40	.4348	.0132	16	.2739
KLF4	.0173	10	.1025	.0072	28	.8142	.0143	21	.2312	.0119	20	.5516
ESR1	.0169	11	.0357	.0106	7	.0941	.0127	27	.0888	.0176	3	<.0001
ELK4	.0168	12	<.0001	.0125	4	<.0001	.0152	16	<.0001	.0086	39	.1318
TFAP2C	.0139	17	.0656	.0114	6	.0941	.0148	19	.037	.0121	19	.2099
PLAG1	.0124	21	.263	.0092	15	.4136	.0219	5	<.0001	.0146	8	.1554
FOXQ1	.0115	28	.9318	.0099	10	.7905	.0209	7	.2846	.0107	27	.927
FOSL1	.0082	57	.9175	.0061	41	.6166	.0220	4	.037	.0131	17	.3496
NFIL3	.0077	62	.2365	.0067	33	.0304	.0264	1	.4669	.0209	1	.7121
FOS	.0068	73	.9175	.0057	48	.5212	.0198	9	.037	.0112	24	.5139
JUNB	.0067	77	.9318	.0059	43	.6392	.0236	2	<.0001	.0146	9	.2299
RFX1	.0019	159	.3532	.0009	164	<.0001	.0233	3	<.0001	.0070	48	.3496
RFX2	.0019	158	.4041	.0012	163	.0482	.0200	8	<.0001	.0049	81	.6245

B Differential gene expression for significantly involved transcription factors.

	ECLIPSE		COPDGene		LGRC		LTCDNM	
transcription factor	dTFI rank	LIMMA p	dTFI rank	LIMMA p	dTFI rank	LIMMA p	dTFI rank	LIMMA p
SP2	1	.1756	9	.6517	6	.0075	2	.0009
E2F4	2	.3913	3	.9367	14	.0878	7	.8232
SP1	3	.3634	18	.0838	10	.4242	4	.9759
ZNF263	4	.9834	16	.0028	11	.0271	6	.1859
EGR1	5	.4379	23	.8540	28	.7979	5	.0378
NRF1	6	.0966	5	.0045	30	.2974	11	.3418
GABPA	7	.4650	2	.5138	12	.3868	32	.5771
ELK1	8	.0913	1	.9010	17	.7968	40	.0005
ZFX	9	.8253	24	.5795	40	.0474	16	.1572
KLF4	10	.1915	28	.0025	21	.0526	20	.1159
ESR1	11	.9598	7	.5853	27	.7246	3	.3477
ELK4	12	.0001	4	.8057	16	.0183	39	.7314
TFAP2C	17	.2318	6	.9574	19	.5853	19	.6754
PLAG1	21	.0384	15	.0008	5	.0371	8	.9523
FOXQ1	28	.4543	10	.5314	7	.0503	27	.5340
FOSL1	57	.5850	41	.6995	4	.8708	17	.3686
NFIL3	62	.0404	33	.1191	1	.7605	1	.8650
FOS	73	.5156	48	.6668	9	.9500	24	.7891
JUNB	77	.0197	43	.9526	2	.3996	9	.6077
RFX1	159	.0361	164	.0885	3	.0175	48	.8285
RFX2	158	.0109	163	.0059	8	.0004	81	.1345

Additional Table 1: **Top Transcription Factor Hits. A** Combined list of transcription factors which were among the top 10 hits (out of 166 available transcription factors) in any of the 4 studies, ordered by the dTFI in the ECLIPSE study. For each study, columns indicate the transcription factor's (1) differential transcription factor Involvement, (2) dTFI Rank within list of transcription factors, (3) and Significance of dTFI by false discovery rate. **B** The same list of top transcription factors evaluated for differential gene expression analysis using LIMMA. A substantial number of differentially involved transcription factors do not exhibit gene expression differentiation, highlighting the ability of MONSTER to identify key features distinguishing phenotypes which are not detectable via gene expression analysis.

[2] Timothy M Bahr, Grant J Hughes, Michael Armstrong, Rick Reisdorph, Christopher D Coldren, Michael G Edwards, Christina Schnell, Ross Kedl, Daniel J LaFlamme, Nichole Reisdorph, et al. Peripheral blood mononuclear cell gene expression in chronic obstructive pulmonary disease. *American journal of respiratory cell and molecular biology*, 49(2):316–323, 2013.

[3] Manhong Dai, Pinglang Wang, Andrew D Boyd, Georgi Kostov, Brian Athey, Edward G Jones, William E Bunney, Richard M Myers, Terry P Speed, Huda Akil, et al. Evolving gene/transcript definitions significantly alter the interpretation of genechip data. *Nucleic acids research*, 33(20):e175–e175, 2005.

[4] Lin S Du P, Kibbe WA. *Using lumi, a package processing Illumina Microarray*, 2007. Bioconductor R package.

[5] Jeremiah J Faith, Boris Hayete, Joshua T Thaden, Ilaria Mogno, Jamey Wierzbowski, Guillaume Cottarel, Simon Kasif, James J Collins, and Timothy S Gardner. Large-scale mapping and validation of escherichia coli transcriptional regulation from a compendium of expression profiles. *PLoS Biol*, 5(1):e8, 2007.

[6] Michele Filosi, Roberto Visintainer, and Samantha Riccadonna. *nettools: A Network Comparison Framework*, 2014. R package version 1.0.1.

[7] Brendan J Frey and Delbert Dueck. Clustering by passing messages between data points. *science*, 315(5814):972–976, 2007.

[8] Laurent Gautier, Leslie Cope, Benjamin M Bolstad, and Rafael A Irizarry. affy-analysis of affymetrix genechip data at the probe level. *Bioinformatics*, 20(3):307–315, 2004.

[9] Kimberly Glass, Curtis Huttenhower, John Quackenbush, and Guo-Cheng Yuan. Passing messages between biological networks to refine predicted interactions. *PloS one*, 8(5):e64832, 2013.

[10] Kimberly Glass, John Quackenbush, Edwin K Silverman, Bartolome Celli, Stephen I Rennard, Guo-Cheng Yuan, and Dawn L DeMeo. Sexually-dimorphic targeting of functionally-related genes in copd. *BMC systems biology*, 8(1):118, 2014.

[11] Kimberly Glass, John Quackenbush, Dimitrios Spentzos, Benjamin Haibe-Kains, and Guo-Cheng Yuan. A network model for angiogenesis in ovarian cancer. *BMC bioinformatics*, 16(1):115, 2015.

[12] Sven Heinz, Christopher Benner, Nathanael Spann, Eric Bertolino, Yin C Lin, Peter Laslo, Jason X Cheng, Cornelis Murre, Harinder Singh, and Christopher K Glass. Simple combinations of lineage-determining transcription factors prime cis-regulatory elements required for macrophage and b cell identities. *Molecular cell*, 38(4):576–589, 2010.

[13] Rafael A Irizarry, Bridget Hobbs, Francois Collin, Yasmin D Beazer-Barclay, Kristen J Antonellis, Uwe Scherf, and Terence P Speed. Exploration, normalization, and summaries of high density oligonucleotide array probe level data. *Biostatistics*, 4(2):249–264, 2003.

[14] W Evan Johnson, Cheng Li, and Ariel Rabinovic. Adjusting batch effects in microarray expression data using empirical bayes methods. *Biostatistics*, 8(1):118–127, 2007.

[15] Peter Langfelder and Steve Horvath. Wgcna: an r package for weighted correlation network analysis. *BMC bioinformatics*, 9(1):1, 2008.

[16] Peter Langfelder and Steve Horvath. Fast R functions for robust correlations and hierarchical clustering. *Journal of Statistical Software*, 46(11):1–17, 2012.

[17] Taotao Lao, Kimberly Glass, Weiliang Qiu, Francesca Polverino, Kushagra Gupta, Jarrett Morrow, John Dominic Mancini, Linh Vuong, Mark A Perrella, Craig P Hersh, et al. Haploinsufficiency of hedgehog interacting protein causes increased emphysema induced by cigarette smoke through network rewiring. *Genome medicine*, 7(1):1, 2015.

[18] Michael Levine and Robert Tjian. Transcription regulation and animal diversity. *Nature*, 424(6945):147–151, 2003.

[19] Daniel Marbach, James C Costello, Robert Küffner, Nicole M Vega, Robert J Prill, Diogo M Camacho, Kyle R Allison, Manolis Kellis, James J Collins, Gustavo Stolovitzky, et al. Wisdom of crowds for robust gene network inference. *Nature methods*, 9(8):796–804, 2012.

[20] Adam A Margolin, Ilya Nemenman, Katia Basso, Chris Wiggins, Gustavo Stolovitzky, Riccardo D Favera, and Andrea Califano. Aracne: an algorithm for the reconstruction of gene regulatory networks in a mammalian cellular context. *BMC bioinformatics*, 7(Suppl 1):S7, 2006.

[21] Anthony Mathelier, Xiaobei Zhao, Allen W Zhang, François Parcy, Rebecca Worsley-Hunt, David J Arenillas, Sorana Buchman, Chih-yu Chen, Alice Chou, Hans Ienasescu, et al. Jaspar 2014: an extensively expanded and updated open-access database of transcription factor binding profiles. *Nucleic acids research*, page gkt997, 2013.

[22] Luca Pinello, Jian Xu, Stuart H Orkin, and Guo-Cheng Yuan. Analysis of chromatin-state plasticity identifies cell-type–specific regulators of h3k27me3 patterns. *Proceedings of the National Academy of Sciences*, 111(3):E344–E353, 2014.

[23] Weiliang Qiu, Dawn L DeMeo, Isaac Houston, Victor M Pinto-Plata, Bartolome R Celli, Nathaniel Marchetti, Gerard J Criner, Raphael Bueno, GRJ Morrow, K Washko, et al. Network analysis of gene expression in severe copd lung tissue samples. In *A30. BIG DATA: HARVESTING FRUITS FROM COPD AND LUNG CANCER*, pages A1253–A1253. Am Thoracic Soc, 2015.

[24] Timothy Ravasi, Harukazu Suzuki, Carlo Vittorio Cannistraci, Shintaro Katayama, Vladimir B Bajic, Kai Tan, Altuna Akalin, Sebastian Schmeier, Mutsumi Kanamori-Katayama, Nicolas Bertin, et al. An atlas of combinatorial transcriptional regulation in mouse and man. *Cell*, 140(5):744–752, 2010.

[25] Erzsébet Ravasz, Anna Lisa Somera, Dale A Mongru, Zoltán N Oltvai, and A-L Barabási. Hierarchical organization of modularity in metabolic networks. *science*, 297(5586):1551–1555, 2002.

[26] Elizabeth A Regan, John E Hokanson, James R Murphy, Barry Make, David A Lynch, Terri H Beaty, Douglas Curran-Everett, Edwin K Silverman, and James D Crapo. Genetic epidemiology of copd (copdgene) study design. *COPD: Journal of Chronic Obstructive Pulmonary Disease*, 7(1):32–43, 2011.

[27] Dave Singh, Steven M Fox, Ruth Tal-Singer, Stewart Bates, John H Riley, and Bartolome Celli. Altered gene expression in blood and sputum in copd frequent exacerbators in the eclipse cohort. *PloS one*, 9(9):e107381, 2014.

[28] Robert Tibshirani. Regression shrinkage and selection via the lasso. *Journal of the Royal Statistical Society. Series B (Methodological)*, pages 267–288, 1996.

[29] David GP van IJzendoorn, Kimberly Glass, John Quackenbush, and Marieke L Kuijjer. Pypanda: a python package for gene regulatory network reconstruction. *arXiv preprint arXiv:1604.06783*, 2016.

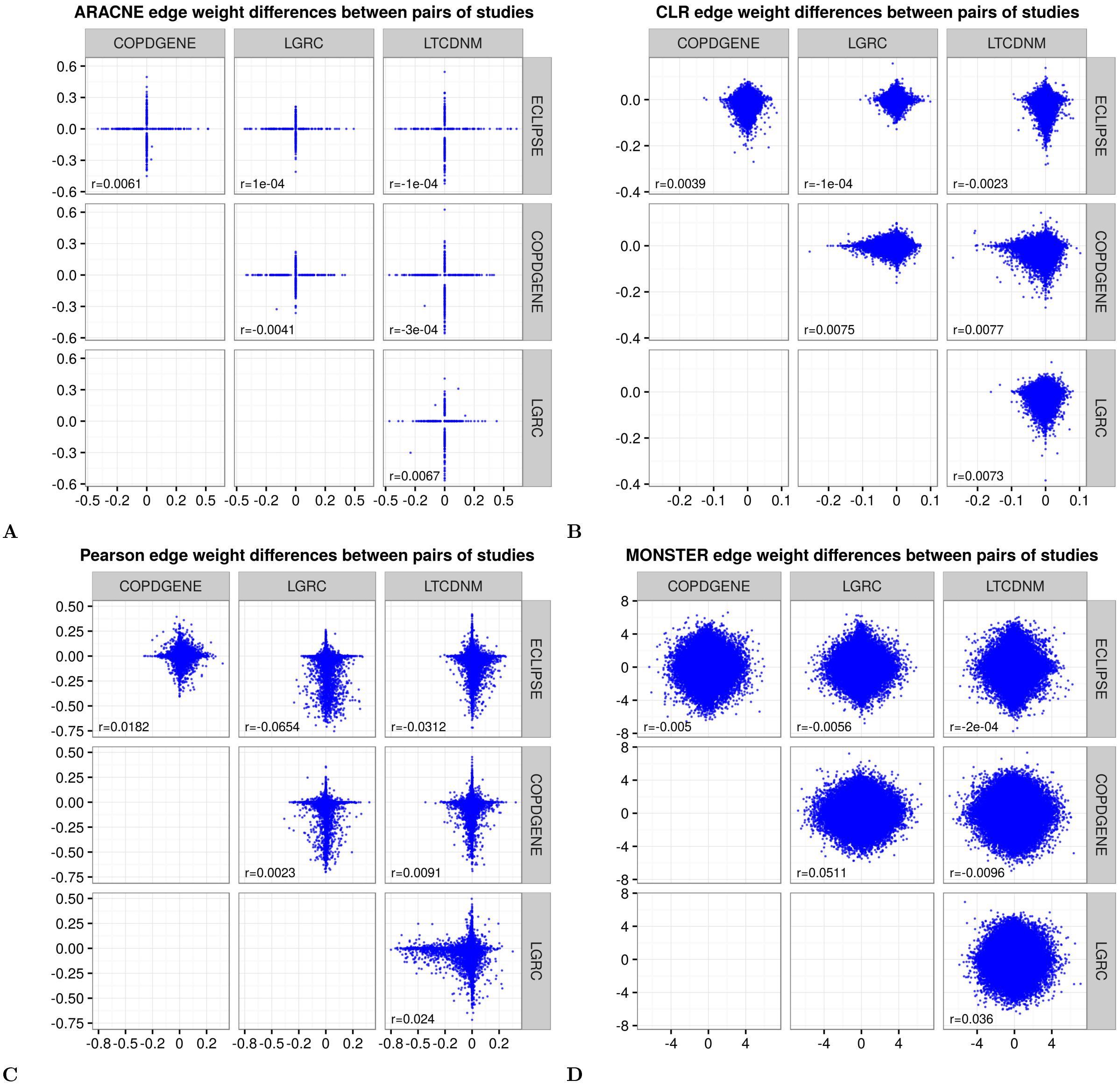

Supporting Figure 3: **Edge weight differences between cases and controls do not correlate across studies.** Using MONSTER and three other commonly used methods, we performed network inference separately on cases and controls in four COPD data sets. Here, the case-control difference is compared for each method in each data set. Most methods had very poor overall concordance in the edge weight differences they estimated. The methods tested were **A** Algorithm for the Reconstruction of Gene Regulatory Networks (ARACNE), **B** Context Likelihood of Relatedness (CLR), **C** Pearson correlation networks, such as in Weighted Gene Correlation Network Analysis (WGCNA), and **D** MONSTER. No detectable agreement between studies exist were found, regardless of network inference method or tissue type.

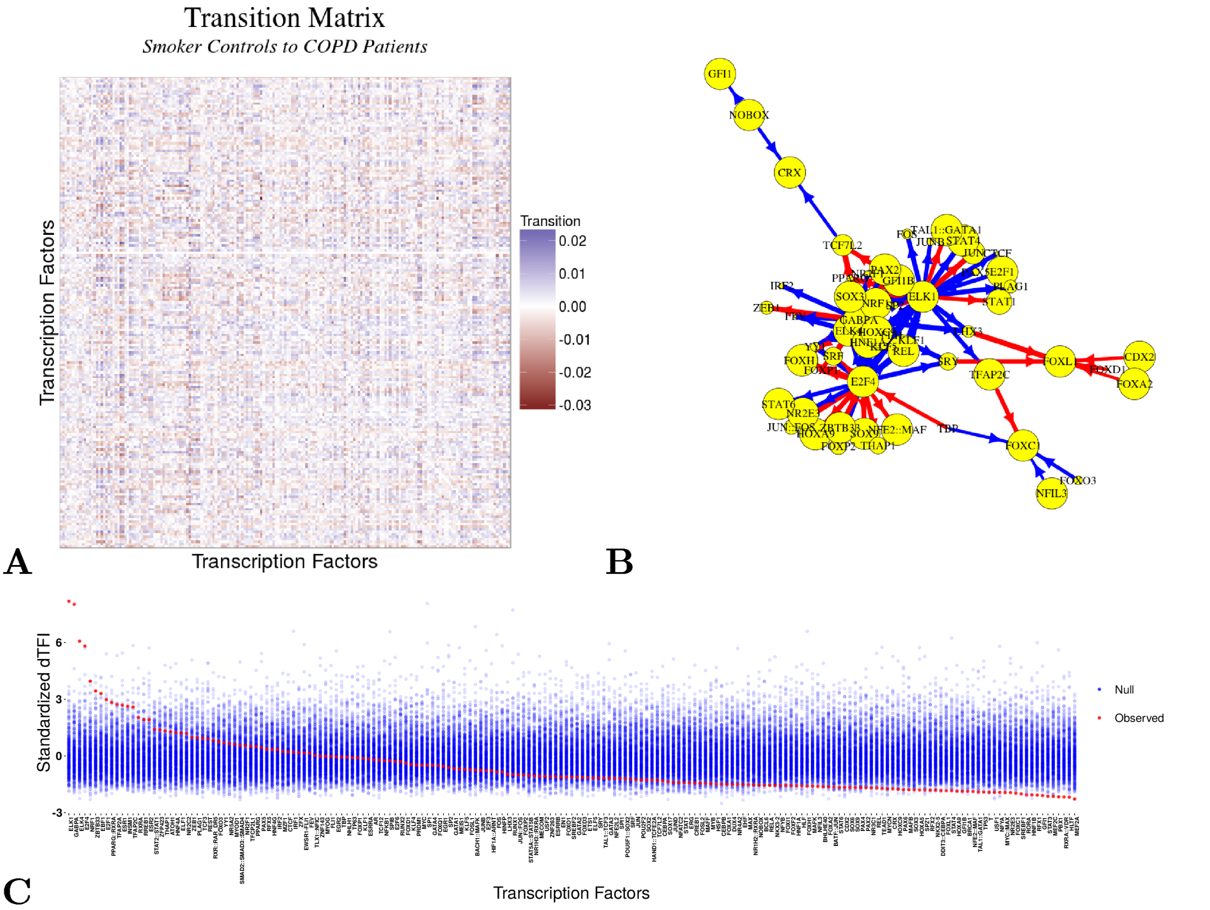

COPDGENE

Additional Figure 1: **MONSTER analysis results for COPDGENE study. A** Heatmap depicting the transition matrix calculated from smoker controls to COPD cases by applying MONSTER to the COPDGene study. For the purposes of visualization, the magnitude of the diagonal is set to zero. **B** A network visualization of the strongest 100 transitions identified based on the transition matrix shown in **A**. Arrows indicate a change in edges from a transcription factor in the Control network to resemble those of a transcription factor in the COPD network. Edges are sized according to the magnitude of the transition and nodes (transcription factors) are sized by the dTFI for that transcription factor. The gain of targeting features is indicated by the color blue while the loss of features is indicated by red. **C** The dTFI score from MONSTER (red) and the background null distribution of dTFI values (blue) as estimated by 400 random sample permutations of the data.

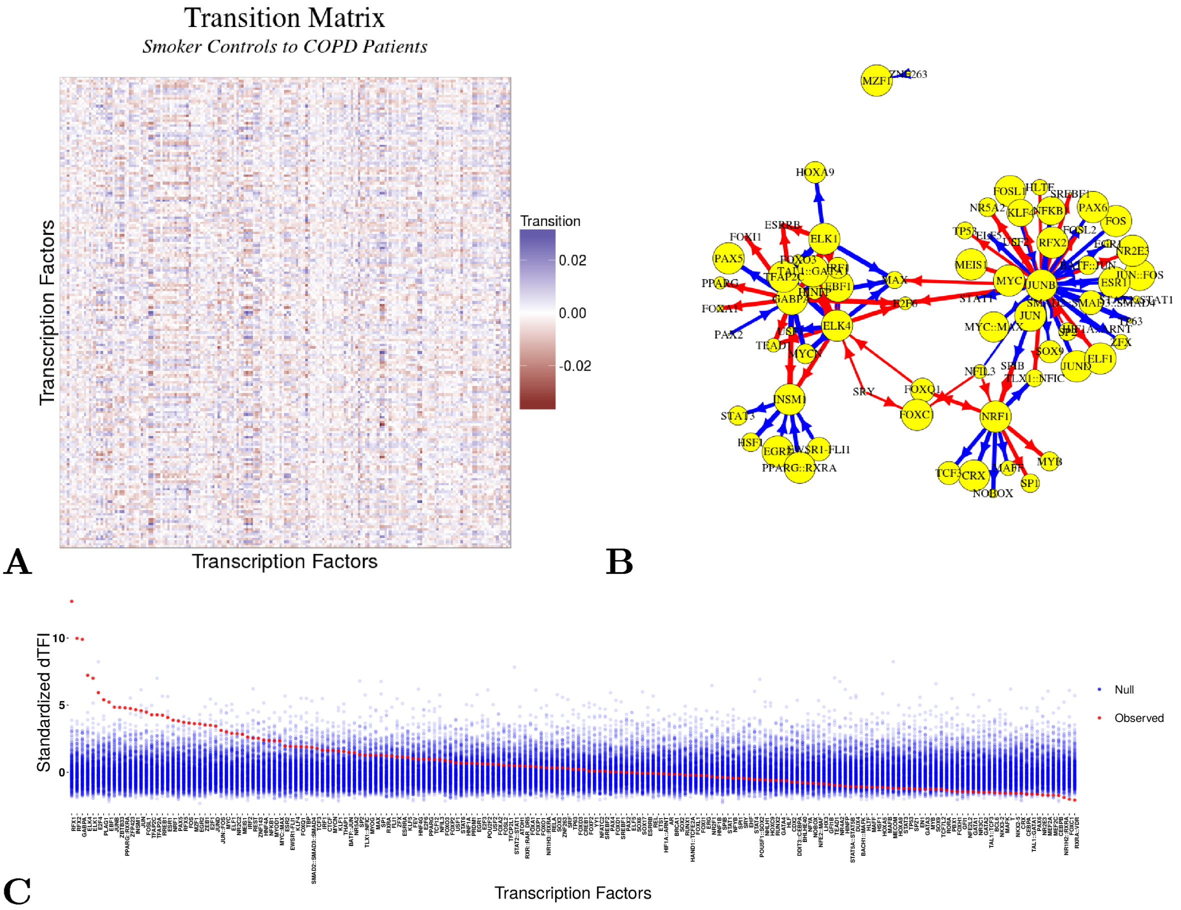

LGRC

Additional Figure 2: **MONSTER analysis results for LGRC study. A** Heatmap depicting the transition matrix calculated from smoker controls to COPD cases by applying MONSTER to the LGRC study. For the purposes of visualization, the magnitude of the diagonal is set to zero. **B** A network visualization of the strongest 100 transitions identified based on the transition matrix shown in **A**. Arrows indicate a change in edges from a transcription factor in the Control network to resemble those of a transcription factor in the COPD network. Edges are sized according to the magnitude of the transition and nodes (transcription factors) are sized by the dTFI for that transcription factor. The gain of targeting features is indicated by the color blue while the loss of features is indicated by red. **C** The dTFI score from MONSTER (red) and the background null distribution of dTFI values (blue) as estimated by 400 random sample permutations of the data.

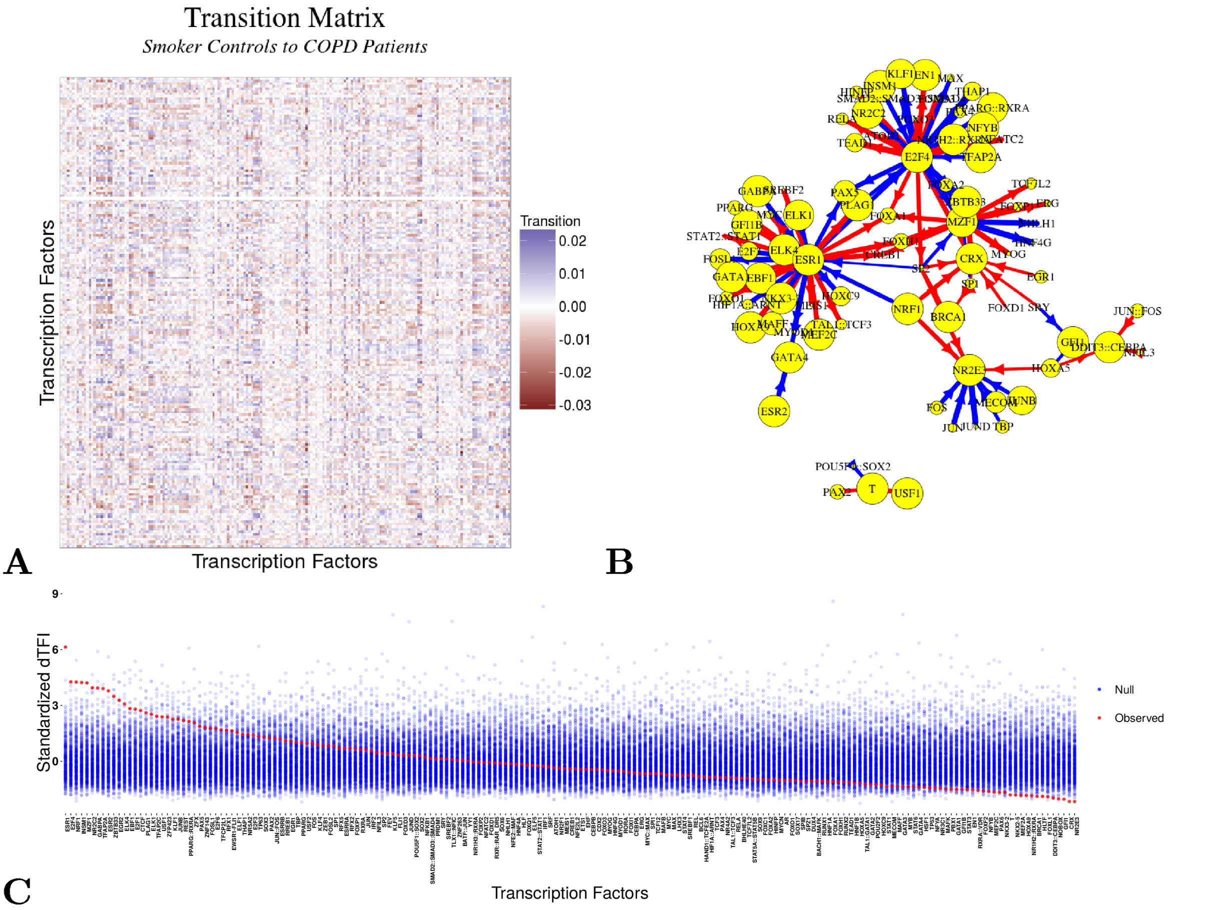

LTCDNM

Additional Figure 3: **MONSTER analysis results for LTCDNM study. A** Heatmap depicting the transition matrix calculated from smoker controls to COPD cases by applying MONSTER to the LTCDNM study. For the purposes of visualization, the magnitude of the diagonal is set to zero. **B** A network visualization of the strongest 100 transitions identified based on the transition matrix shown in **A**. Arrows indicate a change in edges from a transcription factor in the Control network to resemble those of a transcription factor in the COPD network. Edges are sized according to the magnitude of the transition and nodes (transcription factors) are sized by the dTFI for that transcription factor. The gain of targeting features is indicated by the color blue while the loss of features is indicated by red. **C** The dTFI score from MONSTER (red) and the background null distribution of dTFI values (blue) as estimated by 400 random sample permutations of the data.

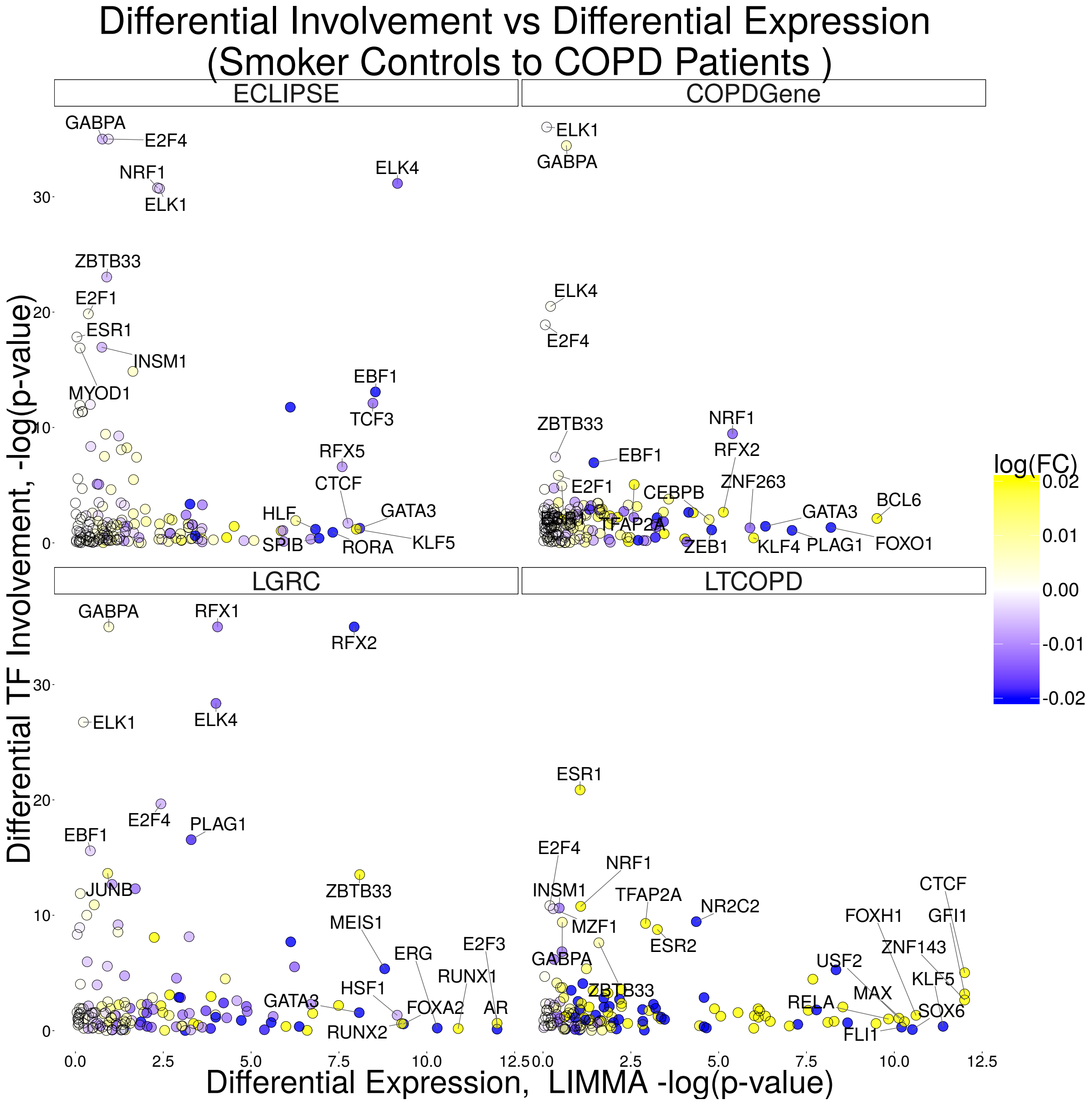

Additional Figure 4: **Differentially transcription factor involvement vs differential gene expression in four studies of COPD.** Plots of the differential expression of transcription factors based on LIMMA, and their different involvement (dTF1) based on MONSTER. We observe much higher consistency between the transcription factors highlighted using MONSTER compared to LIMMA. In addition, we note that MONSTER commonly finds transcription factors which are differentially involved but are expressed at similar levels across cases and controls. This demonstrates the unique potential MONSTER has for discovery beyond standard gene expression analysis.
